# Investigation of the Effect of End Mill-Geometry on Roughness and Surface Strain-Hardening of Aluminum Alloy AA6082

**DOI:** 10.3390/ma13143078

**Published:** 2020-07-10

**Authors:** Pavel Filippov, Michael Kaufeld, Martin Ebner, Ursula Koch

**Affiliations:** 1 Munich University of Applied Sciences, 80335 Munich, Germany; u.koch@hm.edu; 2 Department of Earth- and Environmental Science, Ludwig Maximilian University Munich, 80333 Munich, Germany; 3 Faculty of Mechatronics and Medical Engineering, Ulm University of Applied Sciences, 89075 Ulm, Germany; michael.kaufeld@thu.de; 4 Rohde & Schwarz GmbH & Co. KG, Teisnach Plant, 94244 Teisnach, Germany; Martin.Ebner@rohde-schwarz.com

**Keywords:** micromechanics, indentation, hardness, surface work hardening, aluminum

## Abstract

Micro-milling is a promising technology for micro-manufacturing of high-tech components. A deep understanding of the micro-milling process is necessary since a simple downscaling from conventional milling is impossible. In this study, the effect of the mill geometry and feed per tooth on roughness and indentation hardness of micro-machined AA6082 surfaces is analyzed. A solid carbide (SC) single-tooth end-mill (cutting edge radius 670 nm) is compared to a monocrystalline diamond (MD) end-mill (cutting edge radius 17 nm). Feed per tooth was varied by 3 μm, 8 μm and 14 μm. The machined surface roughness was analyzed microscopically, while surface strain-hardening was determined using an indentation procedure with multiple partial unload cycles. No significant feed per tooth influence on surface roughness or mechanical properties was observed within the chosen range. Tools’ cutting edge roughness is demonstrated to be the main factor influencing the surface roughness. The SC-tool machined surfaces had an average *R_q_* = 119 nm, while the MD-tool machined surfaces reached *R_q_* = 26 nm. Surface strain-hardening is influenced mainly by the cutting edge radius (size-effect). For surfaces produced with the SC-tool, depth of the strain-hardened zone is higher than 200 nm and the hardness increases up to 160% compared to bulk. MD-tool produced a thinner strain-hardened zone of max. 60 nm while the hardness increased up to 125% at the surface. These findings are especially important for the high-precision manufacturing of measurement technology modules for the terahertz range.

## 1. Introduction

Miniaturization of metallic components brings advantages such as space savings, lower energy consumption or improved behavior of high-frequency components (transmission, reflection, etc.). Manufacturing of such parts on industrial scale requires the ability to reliably produce micro-scaled structures. That means, that a manufacturing tolerance on the sub-micron level is necessary. Micro-milling is a widely used technology for machining miniaturized parts with high potential. It presents a technological down-scale of the traditional milling methodology. It also shares the advantages of the macro-milling: high material removal rate and great process flexibility. Hence, it is possible to produce complex three-dimensional geometries with acceptable manufacturing times.

One important application of micro-milling is the manufacturing of waveguides for giga- and terahertz applications. With increasing frequency, the wavelength is approaching the lower μm-range and surface quality become increasingly important. The waveguide efficiency is influenced significantly by the surface roughness. As it is shown by Dikemlik et al. surface roughness significantly influences the reflection and affects the signal-noise ratio [1]. This could be confirmed by Iwai et al. for high-frequency transmission lines along with a method to measure the effect [2]. Tian et al. demonstrated a successful fabrication of a terahertz waveguide with extremely low transmission loss due to, amongst other things, low surface roughness [3]. Thus, precise control of the manufacturing process is necessary to ensure the reliable fabrication of these components.

The micro-milling process can be simply scaled down to some degree. However, new challenges arise on the technological and on the material part when a critical grade of miniaturization is reached. Therefore, the influencing factors and their behavior on microscale must be studied in detail. Numerous factors influence surface integrity, such as tool properties, cutting parameters, workpiece properties, and cutting phenomena, which are summarized by Wang et al. [4]. As has been shown above, surface roughness is especially important for the quality of high-frequency components. Strain-hardening on the other hand can help to improve control over the manufacturing process. For our study, surface roughness and strain hardening are chosen to represent the surface quality.

Various parameters have been shown to influence surface roughness. Yuan et al. reported that the diamond-tool cutting edge radius between 0.3 μm and 0.6 μm has a considerable effect on surface roughness [5]. An experimental study of copper 101 using tungsten carbide micro-endmills by Filitz et al. indicated that surface roughness increases with feed rate [6]. Additionally, Aurich et al. showed that the tilt angle of the main spindle significantly changes material removal on the microscale and influences burr formation as well as surface topography [7]. Gao et al. surveyed different micro-milling parameters of nickel-based single crystal superalloy [8]. He discovered that feed rate, spindle speed, and milling depth have the most influence on surface roughness. From these sources, it can be concluded that cutting edge radius and feed rate (i.e., single tooth feed) have the most influence on micro-milled surface roughness.

Surface strain-hardening depends on the machined material and is difficult to generalize. In this context, the ploughing effect which significantly increases strain-hardening of the surface and the cutting forces at the micro-level should be mentioned. Klocke et al., for example, found that the cutting regime is dominated by the ploughing effect (alternatively micro-milling size-effect) if the uncut chip thickness in on the same order as the tool edge-radius [9]. Already in 1996 it was reported by Yuan et al., that diamond tool sharpness has a significant influence on surface strain-hardening [5]. Lai et al. showed systematically, that the micro-cutter edge radius is the cause for minimum chip thickness *h_min_* [10]. For OFHC-Copper, the tool cutting edge radius of 2 μm, *h_min_* is proposed to be 0.25 times the cutting edge radius. Additionally, they proposed that the micro-milling size-effect is caused by material behavior at the micron level. Additionally, Biermann et al. characterized the influence of *h_min_* on the burr formation [11]. In conclusion, it can be stated that the interplay of the undeformed chip thickness and *h_min_* which is mainly influenced by the cutting edge radius is critical for surface strain-hardening. These factors are supposed to be most relevant to the current study.

The ploughing effect is rarely measured directly from the surface hardness but is rather associated with the increase of cutting forces on the micro-scale. Niu et al. reported that cutting forces increase with increasing tooth feed and cutting edge radius [12], which is in good agreement with the theory. Furthermore, the mentioned study of Lai et al. presents a model to predict the cutting forces taking into account the size-effect by the strain-gradient plasticity [10]. However, it is important to measure these effects directly from the machined surfaces to verify the materials response. As demonstrated by Boehme et al. on austenitic stainless steel, nanoindentation is an effective method to directly investigate critical changes in mechanical properties of micro-milled surfaces [13].

In the present study, the effect of tool geometry (cutting edge radius, cutting edge roughness) and undeformed chip thickness (via single tooth feed) on surface roughness and strain-hardened zone have been investigated. The tool geometry will certainly influence the resulting surface. However, the exact roughness is difficult to predict theoretically. For this reason, surface morphology was studied experimentally.

While the sharper cutting edge will produce less strain-hardening, the depth and hardness of the strain-hardened zone cannot be predicted. Therefore surface strain-hardening by micro-milling has been researched by means of multi-step nanoindentation.

Two different tool geometries with cutting edge radius of 17 nm and 670 nm are compared. Additionally, the undeformed chip thickness is varied via single tooth feed by 3 μm, 8 μm and 14 μm. The roughness of the machined AA6082 aluminum surfaces is examined microscopically. The surface hardness is determined by instrumented indentation with indentation depth from approx. 200 nm to more than 9 μm.

## 2. Materials and Methods

In the present study, a plate of AA6082 (short for EN AW6082-T651, alloy AlSi1MgMn) was used for the milling experiments. The AA6082-plate was machined with two different tools and different feeds per tooth for each tool. Additional reference samples from the AA6082-plate and 99.999% high purity aluminum were electropolished to represent an undeformed material surface. Indentation analysis was performed for milled and electropolished samples. Roughness was only determined on milled samples. An overview of used materials with respective surface treatments is presented in Table 1.

### 2.1. Materials

AA6082 is a medium strength aluminum alloy with an excellent corrosion resistance which is widely used for machining (average hardness 106 HV20, chemical properties in Table 2). This material is compared to recrystallized 99.999% pure aluminum as a base-line reference (average hardness 12 HV5, chemical properties in Table 3).

### 2.2. Tools and Milling Parameters

For comparison, two different commercially available single-tooth micro end-mills were used for micro-milling. These are a solid carbide (SC) end-mill (Sphinx Tools AG, Derendingen, Switzerland) and a monocrystalline diamond (MD) end-mill (Diatec Diamond Technology LLC, Pforzheim, Germany). Both mills are nominally similar with an effective cutter diameter of 500 μm and the cutting length of 1000 μm and 500 μm respectively.

Important surface producing geometrical characteristics of the tools were not available from the datasheets. For this reason, the tools were inspected microscopically. New unused tools were used for the analysis to represent those used to mill the actual surfaces.

**Figure 1:**
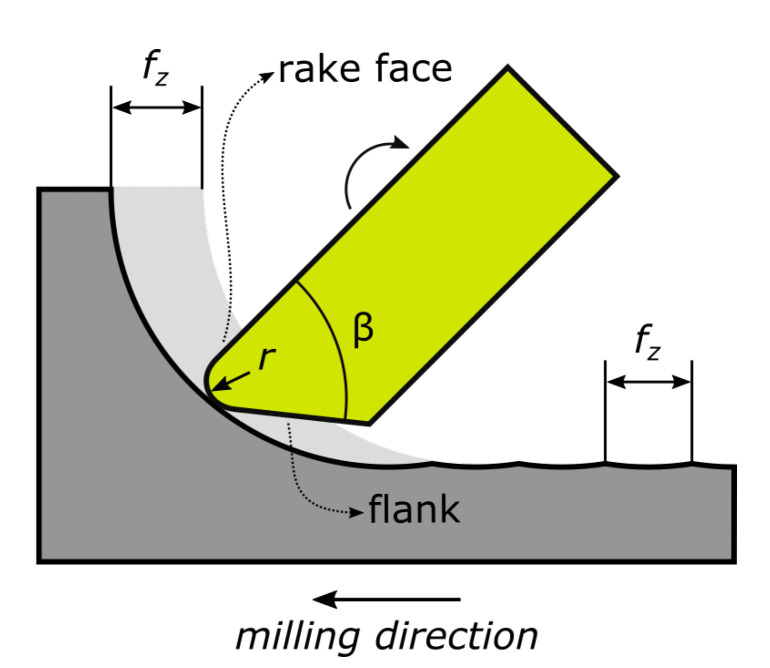
Illustration of the tool parameters wedge angle *β* and cutting edge radius *r* along with single tooth feed *f_z_* and milling direction.

Wedge angle *β*, and cutting edge radius *r* (see Figure 1) were determined directly from optical and Scanning Electron Microscope (SEM) images. Additionally, flank and rake face morphology were evaluated qualitatively.

For cutting edge roughness analysis three optical images of the cutting edge were taken perpendicularly to the rake face (view field 160 μm). Optical images were used preferably to SEM or confocal images, because of the shallow depth of field which generates better edge contrast. Only the inner 80% of the image width were used for analysis because of unsharpness at image edges. Cutting edge profile was extracted via image recognition software (“Analyze_Stripes” plug-in of the Fiji software [14]). From the cutting edge profile one-dimensional roughness values, Root Mean Square Roughness *R_q_* and Maximum peak-to-peak-valley height *R_t_*, were determined according to the ISO4287-1997.

**Figure 2:**
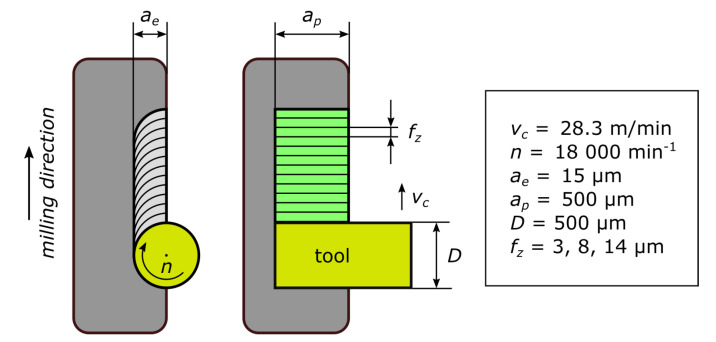
Schematics of the machined structures with corresponding process parameters.

The samples were produced on the “KERN Pyramid Nano” CNC machining center (at Rohde and Schwarz, Teisnach plant, Germany). For all experiments cutting velocity *v_c_*, rotational speed *n*, axial cutting depth *a_e_*, radial cutting depth *a_p_*, and tool diameter *D* were identical, while the feed per tooth *f_z_* was varied by 3, 8 and 14 μm. The process parameters are shown in Figure 2. The peripherally milled surface is used for further analysis (highlighted green in Figure 2).

### 2.3. Electropolishing

To evaluate the effect of micro-milling on surface hardness undeformed (by machining) reference samples were prepared. For this purpose specimens of AA6082 and Al5N were cold mounted and electropolished. These samples represent a non-strain-hardened surface. It has been shown before, that this sample preparation technique removes the deformation layer produces a very smooth aluminum surface [15].

### 2.4. Surface Roughness Analysis

To measure the roughness of the machined surfaces, confocal microscopy images were taken (Leitz Ergoplan confocal microscope, 20× lens). One-dimensional roughness parameters *R_q_* and *R_t_* were determined analogously to cutting edge roughness according to ISO4287-1997 with the Gwyddion software. For these measurements, equidistant profiles (distance of 10 μm) perpendicular (horizontal, 6× per image) and parallel to the milling direction (vertical, 8× per image) were extracted (for directions compare Figure 2).

The analysis of roughness as a result of intermetallic compounds was not the focus of this study. Thus, locations with large intermetallic phases were systematically avoided in roughness measurements. Likewise, locations with imaging artifacts were avoided.

### 2.5. Instrumented Indentation via Enhanced Stiffness Procedure (ESP)

Instrumented indentation is a method widely used to determine material hardness from micro- to the nanoscale. The material is penetrated with a geometrically well-defined indenter to a certain maximum load. Load and indenter displacement (indentation depth) are recorded continuously. Indentation hardness *H_IT_* is then acquired from the contact mechanical analysis of the achieved load-depth-curve. This is usually done with the well established Oliver–Pharr-Method [16] (as proposed by the DIN ISO 14577-1).

This principle is often applied to measure the hardness of thin films, as initially proposed by Bückle [17,18]. Here, knowledge about film thickness is necessary to ensure, that the indentation depth is less than 1/10 of the film thickness (Bückle-rule). To measure *H_IT_* at different depths multiple indents would be necessary.

**Figure 3:**
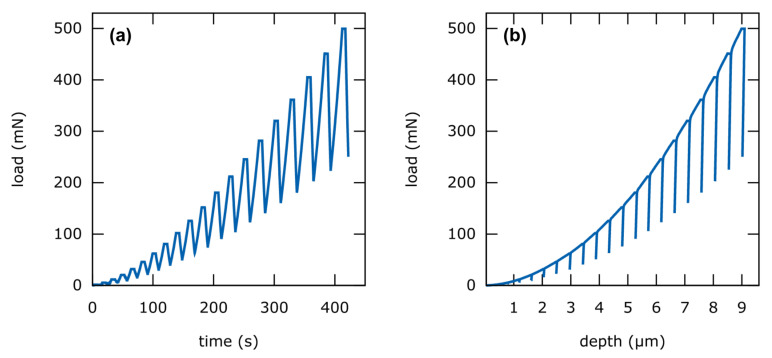
Exemplary curves of an ESP-application. (**a**) programmed load-time-curve. (**b**) resulting load-depth curve.

In this work, we used the so-called Enhanced Stiffness Procedure (ESP) implemented in the Picodentor HM500 (Helmut-Fischer GmbH, Sindelfingen, Germany) which is used for indentation. Here the maximum test load is applied in several partial load cycles each one followed by a partial relief cycle. A time-load diagram of the used ESP-procedure is shown in Figure 3a. The entire measurement is performed at the same location until the maximum test load is reached.

The ESP-load-depth-curve (Figure 3b) is analyzed with the same data analysis model used for single indentation measurements, i.e., Oliver–Pharr method. However, the partial stress-relief-cycles of the ESP-curve are evaluated as separate indentation curves. As a result, *H_IT_* is obtained at different depths from a single indent.

In this study, the Picodentor HM500 equipped with a Berkovich indenter (tip radius 153 nm) is used for the indentation testing. The ESP-procedure is performed with the same parameters for all samples (Table 4). The measurement is repeated at least five times per specimen and an average hardness-depth curve is calculated. The curves are evaluated with the Oliver–Pharr method as described above to achieve indentation hardness values.

### 2.6. Surface Strain-Hardening Analysis

Indentation hardness is an averaged value from a plastically deformed volume below the indenter [19]. Therefore, hardness measured at a certain depth is not the actual hardness at this depth. To achieve additional information about the strain-hardened layer due to micro-milling (i.e., bulk hardness, the hardness of the strain-hardened layer, layer thickness, etc.) a film-substrate model from Equation (1) was fitted to the hardness-depth data.

There are many approaches to model composite hardness of a film-substrate system. Broitman or Fischer–Cripps both show a summary of important models [20,21]. The approach developed by Korsunky et al. is a film-substrate model based on a volume law of mixtures [22]. The advantage of the Korsunsky-model lies in its suitability for numerical fits, which i.e., was demonstrated by Tuck et al. [23,24]. That makes this model also especially suitable for our study.

The effective hardness *H_eff_* (i.e., *H_IT_* measured directly by nanoindentation) is described by Korsunsky et al. as the function of film hardness *H_f_* and substrate hardenss *H_s_*:

(1) $$H_{eff}=H_{s}+\frac{H_{f}-H_{s}}{1+\frac{t}{\alpha }{\left(\frac{h}{t}\right)}^{2}}$$

Here, *t* is the film thickness, *h* the indentation depth, and *α* the factor which describes film plasticity. This model assumes constant hardness over depth within the surface layer. In our case it means, that the strain-hardened zone is modeled with constant hardness over depth. Under this prerequisite, the Korsunsky-model was numerically fitted to the averaged hardness depth curves achieved from the ESP-measurements. This was done by the nonlinear least-squares Marquardt–Levenberg algorithm as implemented in the Gnuplot fit module (Gnuplot 5.2: an interactive plotting program).

## 3. Results

### 3.1. Tool Geometry

**Figure 4:**
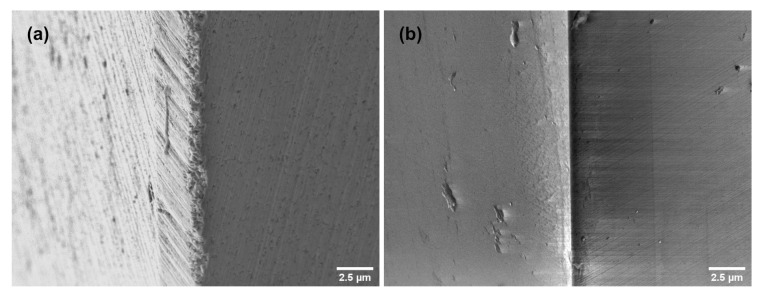
SEM-image on the flank (**left**) and rake (**right**) face of (**a**) SC-tool. (**b**) MD-tool.

Exemplary SEM-images of the cutting edge in Figure 4 show flank and rake face of both mills for comparison. The cutting edge of the SC-tool (Figure 4a) shows grinding traces on the rake face and the flank. Additionally, embedded tungsten carbide particles can be distinguished in the cutting edge. In contrast to this the MD-tool (Figure 4b) appears very smooth, almost featureless. The visible contamination is insignificant for the quality of the milling process.

The wedge angle of both tools was determined from optical microscope images. While the SC-tool has an acute wedge angle (approx. 54°), the wedge angle of the MD-tool is almost a right angle (approx. 83°).

**Figure 5:**
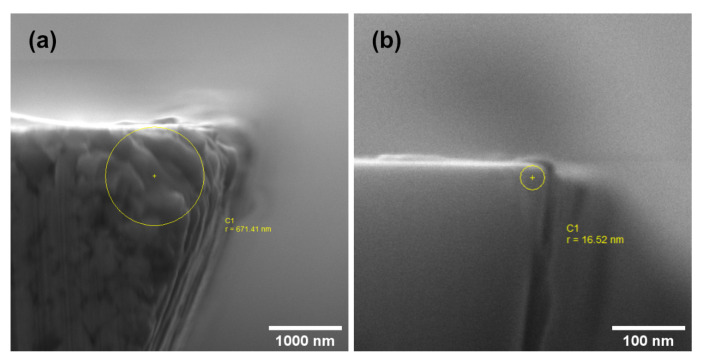
SEM-images of the cutting edge radius, taken parallel to the tool axis. (**a**) SC-tool. (**b**) MD-tool.

Cutting edge radii approximated on the basis of the SEM images shown in Figure 5 are 671 nm and 17 nm for the SC-tool and MD-tool respectively. The image of the MD-tool (Figure 5b) is magnified ten times as much as the image of the SC-tool (Figure 5a). Yet, it was challenging to determine the cutting edge radius of the MD-mill because it is extremely small. The radius of the SC-tool is 35 times larger than the MD-tool radius.

**Figure 6:**
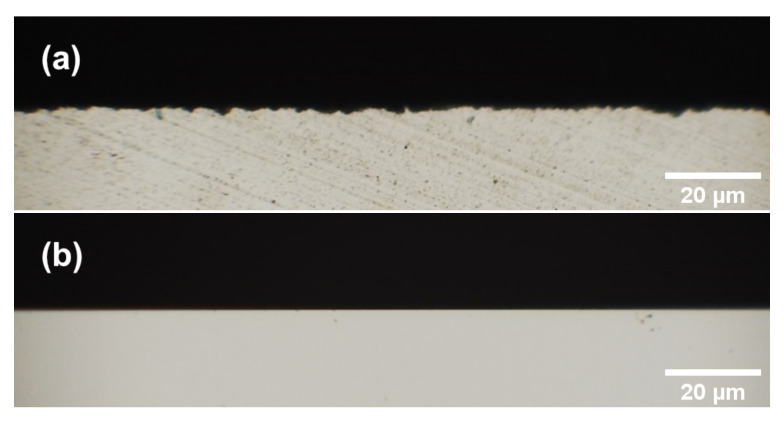
Optical images of the cutting edges for the roughness evaluation. The images are taken from the direction of the rake face. View field for all images is 160 μm. (**a**)SC-tool. (**b**) MD-tool.

The SC-tool has a measurable roughness (Figure 6a), but it is hard to recognize any features along the cutting edge of the MD-tool (Figure 6b). Subsequently, *R_q_* and *R_t_* are determined based on these images. The resulting roughness values are shown in Table 5. It is remarkable, that the cutting edge roughness *R_q_* of both tools differ by more than a factor of 25. Indeed, the cutting edge of the MD-tool is so smooth, that the resulting *R_q_* and *R_t_* values probably show the measurement error instead of the actual roughness.

At this point, it can be concluded that the cutting edge radii of both mills are significantly different. The cutting edge radius of the SC-tool is still very sharp (*r* ≈ 670 nm) in comparison to conventional mills. In contrast, *r* of the MD-tool is difficult to determine even with electron microscopy. The same applies to the cutting edge roughness. Here the *R_q_* of the SC-tool is approx. 2400 nm with the roughness of the MD-tool being not measurable. Other determined characteristics (wedge angle, rake face, and flank morphology) are not regarded as relevant for the interpretation of the results.

### 3.2. Morphology of the Machined Surfaces

**Figure 7:**
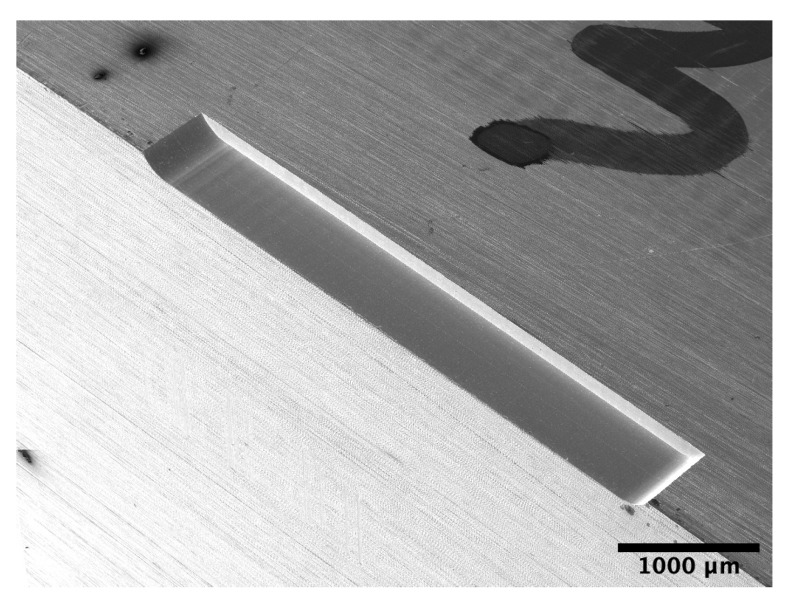
Example of the produced structures. The roughness and indentation measurements have been performed on the horizontal surface.

The micro-milled structure is shown in Figure 7. The measurements are performed in the middle of the horizontal surface.

**Figure 8:**
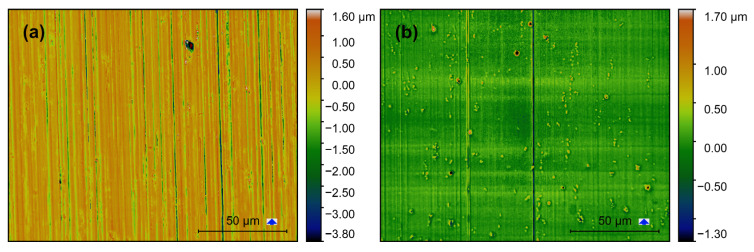
Confocal images of AA6082 surfaces milled with *f_z_* = 3 μm: (**a**) SC-tool, (**b**) MD-tool.

**Figure 9:**
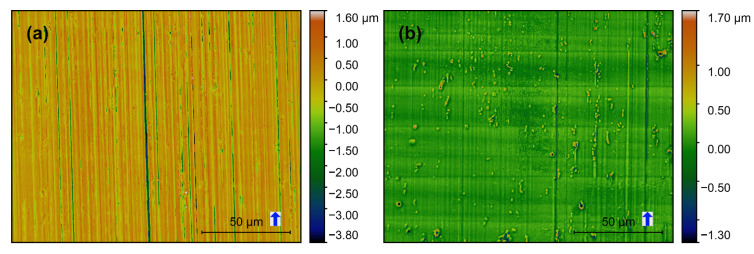
Confocal images of AA6082 surfaces milled with *f_z_* = 8 μm: (**a**) SC-tool, (**b**) MD-tool.

**Figure 10:**
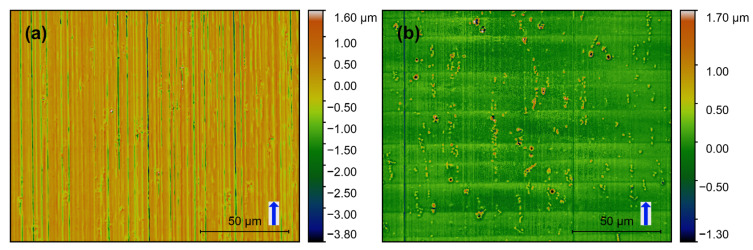
Confocal images of AA6082 surfaces milled with *f_z_* = 14 μm: (**a**) SC-tool, (**b**) MD-tool.

Confocal images of the produced surfaces are shown in Figure 8, Figure 9 and Figure 10. The respective *f_z_* is represented with the blue arrow next to the scale bar, which also indicates the milling direction. Based on these images surface features can be categorized concerning the orientation of milling direction on the image. Height variations parallel and perpendicular to the milling direction are described as “vertical roughness” and “horizontal roughness” respectively. The terms “vertical” and “horizontal” describe the direction in which the one-dimensional roughness is measured (*R_q_*, *R_t_*).

Vertical roughness is mainly defined by *f_z_* which should be visible in the form of lines perpendicular to the milling direction. Horizontal roughness is mainly ruled by the mill’s cutting edge roughness. Additionally, there are non-directional features, namely the intermetallic phases and other inclusions in the base material.

Vertical grooves are observable on all images and indicate the milling direction. Deep vertical grooves are evident on the SC-tool machined surfaces (Figure 8a–Figure 10a). At the same time, only a few much flatter grooves can be observed on the MD-tool machined surfaces (Figure 8b–Figure 10b). This difference is also reflected in the horizontal roughness (Figure 12). Here the resulting *R_q_* and *R_t_*-values of the MD-mill produced surfaces are almost five times lower than those for the SC-tool.

For all surfaces produced with the same tool (SC or MD), no significant dependency on the *f_z_* could be determined. The *f_z_*-steps can be seen perpendicular to the milling direction on the images produced with the MD-tool (Figure 8b–Figure 10b). These features are unrecognizable on the SC-mill produced surfaces (Figure 8a–Figure 10a).

**Figure 11:**
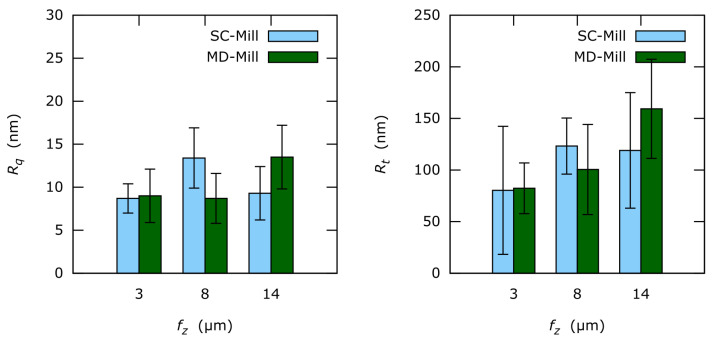
Vertical one-dimensional roughness *R_q_* and *R_t_* of the analyzed surfaces.

**Figure 12:**
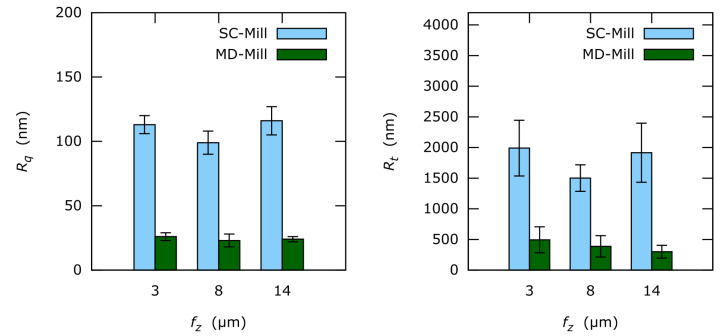
Horizontal one-dimensional roughness values *R_q_* and *R_t_* of the analyzed surfaces.

Line roughness measured parallel to the milling direction (i.e., vertical roughness) is represented in Figure 11. Both *R_q_* and *R_t_* are almost equal regardless of the used tool. Yet, *R_t_* seems to correlate with *f_z_* at least for MD-milled surfaces. The achieved vertical roughness values are much lower compared to the horizontal roughness seen in Figure 12.

Only slight influence of undeformed chip thickness on vertical roughness and no effect on horizontal roughness could be observed for *f_z_* = 3 μm to 14 μm.

### 3.3. Depth Dependent Surface Hardness

**Figure 13:**
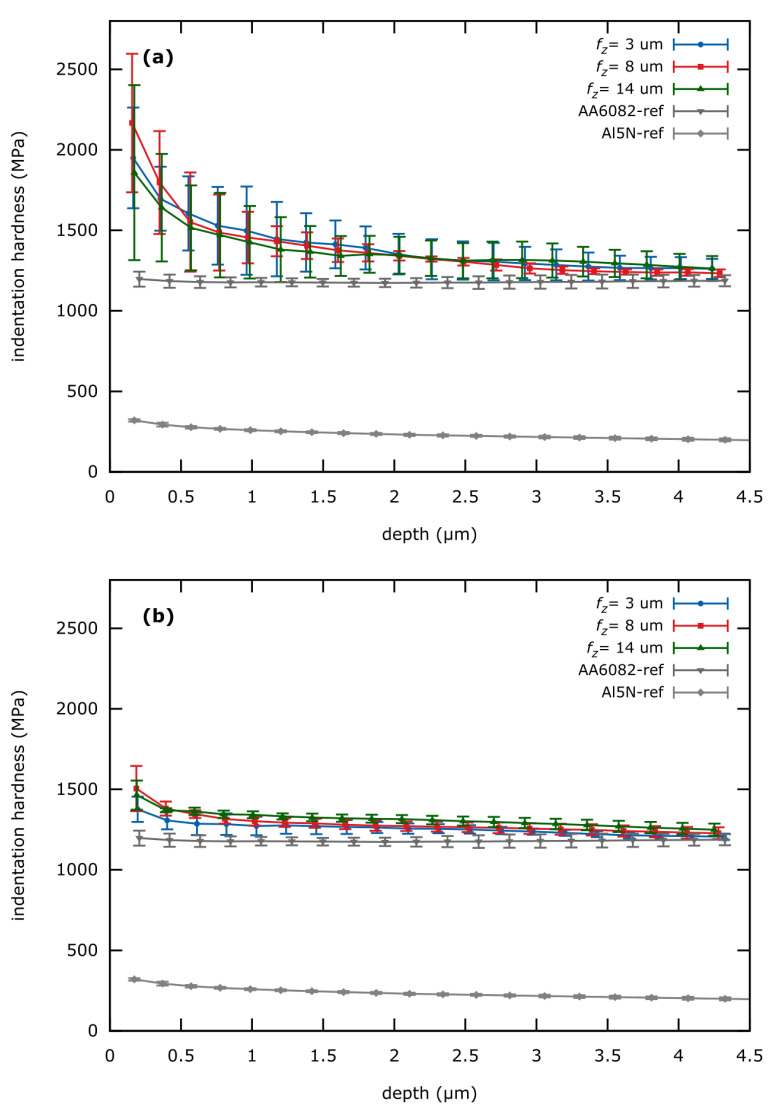
Hardness-depth-curves of machined surfaces compared to electropolished AA6082 and Al5N surfaces as a reference. (**a**) SC-tool machined surface (**b**) MD-tool machined surface.

The depth-dependent hardness data achieved from the ESP-curves are shown in Figure 13. Additionally, min. and max. *H_IT_* values along with an 8%-onset depth (depth at which hardness increases by 8% relative to the bulk value at max. indentation depth) are summarized in Table 6. *H_IT,min_* of all AA6082 samples (milled or electropolished) converge to a value of approx. 1250 MPa at higher indentation depths (Table 6). This value also corresponds with the overall average *H_IT_* of the electropolished AA6082 reference and represents the bulk hardness of the AA6082-alloy.

The electropolished Al5N sample is the weakest with *H_IT_* ≈ 240 MPa, while this value increases at lower indentation depths due to the indentation size effect (ISE). The AA6082 reference has an almost constant *H_IT_* ≈ 1195 MPa at all indentation depths with no signs of the ISE.

As expected, all milled surfaced show strain-hardening in form of *H_IT_* increase at low depths. This effect is especially prominent on SC-tool machined surfaces (Figure 13a). Here the 8%-onset of hardness is at the indentation depth of 2.5 μm. Maximum *H_IT_* of more than 2000 MPa is reached at lowest indentation depths (Table 6).

Strain hardening is much less pronounced for MD-milled surfaces, but still distinguishable in comparison to the AA6082 reference (Figure 13). The 8%-onset is at a lower depth of approx. 500 nm and reaches a maximum *H_IT_* of about 1500 MPa.

The min. and max. values of *H_IT_* together with the 8%-onset depth are summarized in Table 6. Variations of *f_z_* do not appear to influence the strain-hardening. However, this effect is difficult to assess from the ESP-curves only.

### 3.4. Film-Substrate-Model Fit

**Figure 14:**
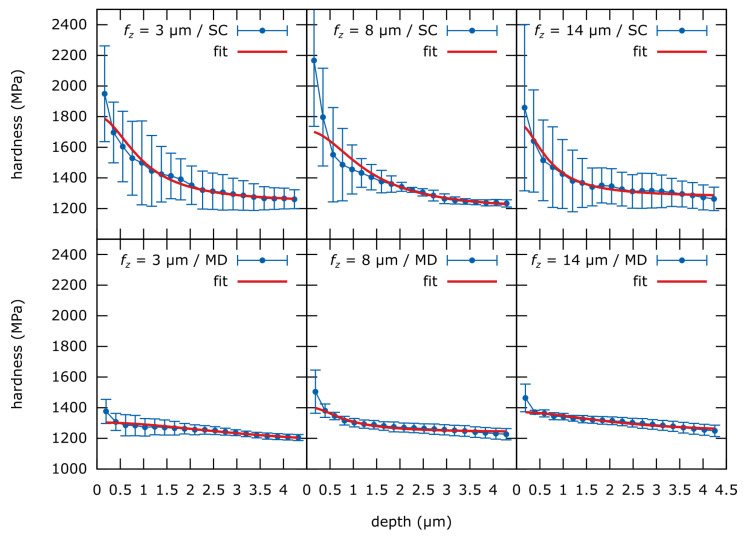
Korsunsky film-substrate-model from Equation (1) [22] fitted to to the averaged ESP-data sets shown in Figure 13.

When fitted to the ESP-curves presented in Figure 14 at first an acceptable fit of the Korsunsky-model from Equation (1) for indentation depth above approx. 500 nm is observed. However, data points at lower depth do not fit the model. Nonetheless, the fitted parameters seem adequate (Table 7). Especially the *H_s_* and *H_f_* values are in the expected range.

## 4. Discussion

### 4.1. Roughness of the Micro-Milled Surfaces

Results demonstrate significant differences for one-dimensional horizontal roughness (perpendicular to the milling direction) of both mills. The surface features are generated by the cutting edge roughness. Cutting edge roughness is replicated on the machined surface, while the original roughness is smoothed out significantly during milling.

In the case of the SC-tool, the cutting edge geometry replicates on the sample surface and produces comparably high roughness with random features (probably due to tungsten carbide particles). Due to this process, the cutting edge *R_q_* is lowered by a factor of 20. Thus, the remaining surface roughness is still remarkable. Here the roughness is mainly influenced by the cutting edge geometry.

On the other hand, the MD-tool produces very smooth surfaces with systematic features. The replication factor of cutting edge roughness is difficult to estimate because the cutting edge roughness of the MD-tool is very low, and could not be determined optically or with SEM. Since the MD-tool cutting edge is extremely smooth, the remaining low surface roughness is mainly causes by two factors: small intermetallic phases (non-directional) and *f_z_* (parallel to the milling direction).

Additionally, systematic vertical lines are visible on confocal images of the MD-milled surfaces. These are generated by the few defects of the MD-tool cutting edge. SEM-images (not shown here) confirm, that these features run through the complete milled surface.

Large intermetallic phases produce height differences much higher, than the effect of the mill geometry (Δ*h* > 10 μm). This poses a potential problem during coating of the so produced component. However, this only can be dealt with by exchanging AA6082 by another purer material with less intermetallic phases.

At least, *f_z_*, *β* and flank morphology have no significant influence on surface roughness. In case of *f_z_* the reason is probably the chosen parameter range.

### 4.2. Surface Strain-Hardening

According to Filitz et al. the specific cutting energy increases with decreasing undeformed chip thickness due to micro-milling size effect [6]. In our terms, this means the that size effect should increase when *f_z_* (corresponding to undeformed chip thickness) converges to *h_min_*. Kim at al. suggest the *h_min_* ≈ *r*/3 [25].

Both mills investigated in this study have different *r*-values and therefore different *h_min_*. For the MD-tool, *f_z_* = 3 μm, which is 500 times greater than the corresponding *h_min_*. This *h_min_*-value is beyond reach (<10 nm), which means, that in case of the MD-tool the milling process is in a pure cutting regime.

This is consistent with the ESP-results where only minimal hardness increase at depths below 500 nm can be observed. This hardness increase can be attributed to surface strain-hardening and not the indentation size effect by the comparison to the electropolished AA6082 reference. Here no significant indentation size effect, i.e., no hardness increase at low depths is observable.

This is however different for the SC-tool. Estimated from the cutting edge radius, the *f_z_* = 3 μm here is only 15 time *h_min_*. Therefore, undeformed chip thickness is much closer to *h_min_* and a significant ploughing effect can be anticipated. This is verified by the achieved results, which demonstrate significant strain-hardening (hardness increase) at depth below 1 μm.

The Korsunsky film-substrate model expects hardness at low indentation depth to be dominated by the film only. Therefore, a plateau at low depths is expected, which is also demonstrated by Tuck [23,24]. This behavior cannot be observed in the present data set (Figure 14). Apparently, the hardness increases with decreasing indentation depth, while the maximum (which would be indicated by a plateau) could not be measured.

**Figure 15:**
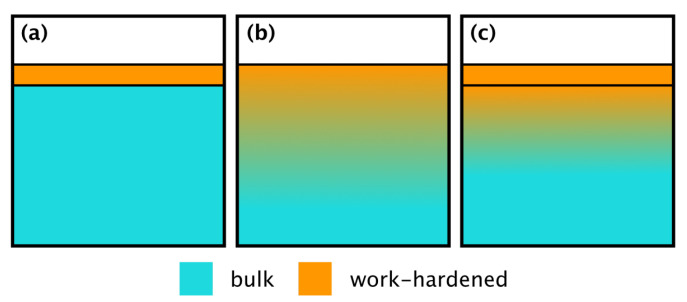
Different interpretations of the indentation results. (**a**) Thin, uniformly hardened film. (**b**) Gradually strain-hardened layer. (**c**) Combination of a gradually strain-hardened layer and a thin film.

As it has been mentioned above, indentation hardness is averaged from the plastic zone beneath the indenter [19]. According to the Bückle–Rule, this zone extends in the indentation direction for least 10 times the indentation depth [17]. This leads to three options for interpreting the experimental data. The different possibilities are illustrated in Figure 15, while all options would lead to no observable hardness plateau at low depths.

Figure 15 presents principal possibilities of strain-hardening at the material surface. The simplest model is a uniformly hard surface layer on top of softer bulk material (Figure 15a). This model also corresponds with the film-substrate-hardness model of Korsunsky [22]. The next possibility is a gradually hardened surface (Figure 15b). Here the maximum hardness at the surface decreases continuously towards the bulk. The last is the combination of the first two models which implies a uniformly hard surface layer and gradually decreasing hardness below it (Figure 15c).

The material stress under the tool has been analyzed for micro-milling by Lai et al. [10]. They demonstrate by FEM-simulations that stress in the material decreases with depth. Since material stress correlates with strain-hardening, the model of a uniformly harden surface layer (Figure 15a) can be discarded.

The model of the gradually hardened surface layer (Figure 15b) corresponds well with the stress-analysis by Lai et al., however, it is lacking the native surface oxide layer. This surface layer could be implemented in the combined model (Figure 15c). However, according to Evertsson et al. the thickness of such layers usually below 10 nm [26]. Such a thin layer would be insufficient to significantly increase surface hardness on the observed length scale and can, therefore, be neglected. Consequently, the model (c) could be practically reduced to the model (b).

No suitable data model is available at the moment. Based on the presented Korsunsky-fits and stress data from the literature it can be concluded that the surfaces generated by micro-milling are gradually strain-hardened. It also can be approximated from the 8%-onset of the hardness increase and the Bückle–Rule, that the hardened layer is very thin. For an approximation of the strain-hardened layer thickness, the average height of the 8%-onset can be divided by 10 according to the Bückle–Rule. This results in a layer thickness of less than 200 nm for the SC-tool- and less than 70 nm for the MD-tool -produced surfaces.

The variation of the *f_z_* seems to have no effect of strain-hardening. However, it should be noted that the *H_IT_*-error is especially high for low depths. The reason is that surface roughness increasingly influences the results at a lower depth. DIN ISO 14577-1 proposes the following relation of the minimum indentation depth and roughness of the analyzed surface: *h* ≥ 20·*R_a_*. This results in a minimum indentation depth *h* of 340 nm for the MD-tool and 1380 nm for the SC-tool. Hardness values achieved for depth below these values are beyond the recommendation of the normative and have to be interpreted cautiously. Therefore, a different approach is needed, to determine the hardness values especially of the SC-tool machined surfaces at very low depth.

## 5. Conclusions and Outlook

Concerning the morphology of micro-machined surfaces, it could be concluded that surface roughness is mainly influenced by the tool geometry and not by the process parameters (in the chosen parameter range). The SC-tool with higher *r* and rough cutting edge replicates on the micro-milled material. The resulting surfaces show high roughness perpendicular to the milling direction (along cutting edge) with no systematic effects parallel to the milling direction. The MD-tool with an extremely small *r* and almost featureless cutting edge produce very smooth surfaces. However, the existent features are systematic and originate from intermetallic phases of all sizes, *f_z_*, and smaller cutting edge defects. Additionally, a slight correlation of *f_z_* to vertical roughness could be observed. These findings are to be taken into account in the selection of materials, tools, and production methods for the manufacture of micro components where surface roughness is critical. This also applies to the production of RF components for use in the terahertz range.

By extracting depth-dependent *H_IT_* of the micro-machined surfaces, much could be learned about the mechanical properties of these surfaces. The main factor influencing the surface strain-hardening is *r* of the used tool. The influence of the process parameters could not be evaluated due to high error at low indentation depths. The Korsunsky model of a uniformly hard film (i.e., strain-hardened layer) is suitable for determining hardness at greater indentation depths. However, for *h* < 500 nm the fit quality is insufficient. This indicates, that the produced surfaces are probably gradually hardened with max. *H_IT_* on the surface.

The hardness gradient and max. *H_IT_* depend highly on *r* and *f_z_* (i.e., undeformed chip thickness). The influence of *f_z_* on strain-hardening could not be observed in this study, because of the chosen parameter range. For SC-tool machined surfaces with big *r* the strain-hardened zone is thinner than 200 nm. For MD-milled surfaces with an almost infinitely small *r* this zone is thinner than 40 nm.

To improve the data analysis of the indentation results a new model has to be developed to incorporate the depth-dependent hardness of the strain-hardened layer at low depth. To study the influence of the process parameters, the experiments should be repeated with different *f_z_*-values. Values below, equal or slightly above *h_min_* depending on the used tool’s *r* are of major interest. Additionally, the effect of the cutting edge wear is still an open point and should be inspected closely.

## Figures and Tables

**Table 1:** Sample overview including the respective raw material, surface treatment, tool used for milling, and feed per tooth.

Material	Surface Finish	Tool	Single Feed Per Tooth
			3 μm
AA6082	Milled	SC	8 μm
			14 μm
			3 μm
AA6082	Milled	MD	8 μm
			14 μm
AA6082	Electropolished	-	-
Al5N	Electropolished	-	-

**Table 2:** Chemical properties of AA6082; provided by the manufacturer (ALRO S. A, Slatina, Romania).

Element	Si	Fe	Cu	Mn	Mg	Cr	Ni	Zn	Ti	Ga	V
Content (wt.%)	1.05	0.47	0.09	0.66	0.97	0.17	0.016	0.076	0.026	0.011	0.017

**Table 3:** Chemical properties of Al5N; provided by the manufacturer (HMW Hauner GmbH & Co. KG, Röttenbach, Germany, obtained by Optical Emission Spectroscopy).

Element	Ca	Fe	Mn	Si	Cr	Mg	Na	Ti	Zr
Content (wt. ppm)	<0.02	0.83	0.056	1.51	0.049	0.73	<0.005	0.067	0.013

**Table 4:** Parameters used for the ESP procedure.

Parameter	Value
Max. test load	500 mN
Total time	422.3 s
True increase time	60 s
Creep	5 s
Unloading	50% of *F_max_*
Number of unloadings	20

**Table 5:** Average values of the cutting edge roughness *R_q_* and *R_t_* for the SC- and MD-tool with the standard deviation *σ*.

Tool	*R_q_* ± *σ* (nm)	*R_t_* ± *σ* (nm)
SC-tool	2355±1551	8881±5208
MD-tool	90±48	661±363

**Table 6:** Summary of the minimum (at max. depth) and maximum (at min. depth) hardness values and the onset depth of the strain-hardening for all machined samples. Average onset values are presented in the table.

Tool	*f_z_*	*H_IT,min_* (MPa)	*H_IT,max_* (MPa)	8%-Onset Depth (μm)
	3	1261±62	1949±313	2.03	
SC-tool	8	1233±24	2166±430	2.26
	14	1262±77	1858±543	1.62	
	3	1205±20	1376±79	0.61	
MD-tool	8	1227±37	1505±141	0.81
	14	1249±37	1464±90	0.60	
AA6082-ref		1187±35	1197±46	-

**Table 7:** Fitted parameters of the Equation (1) fitted to the depth-hardness data from Figure 13, as plotted in Figure 14. The parameters represent the direct output of the plotting software, each with the respective “asymptotic standard error”. For each data set the final sum of squares *S* is shown additionally.

Tool	*f_z_* (μm)	*H_f_*	*H_f,err_*	*H_s_*	*H_s,err_*	*t*	*t_err_*	*α*	*α_err_*	*S*
	3	1236.0	13.3	1803.2	54.1	0.2	82.5	0.2	90.9	0.54
SC	8	1184.3	16.8	1707.5	114.4	0.3	141.4	0.2	82.7	2.70
	14	1275.0	8.8	1764.2	100.4	0.7	393.7	1.5	845.8	0.37
	3	1141.8	102.2	1303.7	10.6	1.3	1552.1	0.1	131.7	1.24
MD	8	1239.4	7.4	1406.0	36.9	0.5	348.7	0.7	487.7	1.79
	14	1235.7	39.0	1371.6	6.1	2.4	1922.8	0.5	406.2	2.34
